# Mithramycin interacts with core histones and modulates epigenetic modifications

**DOI:** 10.1186/1756-8935-6-S1-P106

**Published:** 2013-04-08

**Authors:** Amrita Banerjee, Chandrima Das, Dipak Dasgupta

**Affiliations:** 1 Biophysics Division, Saha Institute of Nuclear Physics, Block-AF, Sector-1, Bidhan Nagar, Kolkata – 700064, West Bengal, India

## Background

Mithramycin (MTR), a clinically approved DNA-binding antitumor antibiotic has been found to cross the blood-brain barrier and is in its preclinical trials in Huntington’s disease (HD) [1]. It improved altered nucleosome homeostasis in HD mice, normalizing the chromatin pattern. It has the ability to rebalance epigenetic histone modifications. The binding properties of MTR with histone proteins have been examined from the perspective of the current proposition from our laboratory to classify small DNA binding molecules in terms of their ability to bind chromosomal DNA alone (single binding mode) or both histones and chromosomal DNA (dual binding mode) [2,3].

## Materials and methods

Chemicals were from Sigma, histones from New England Biolabs, antibodies from Abacm, Active Motif. Spectrofluorimetry, circular dichroism (CD) studies, *in vitro* histone acetyl transferase (HAT) assay and western blot analysis were employed as experimental tools.

## Results

Spectrofluorimetric measurements and CD studies show that MTR binds with micromolar dissociation constant to core histone assembly and its individual histone components. A single isoelliptic point in the CD spectra indicates formation of one type of complex between MTR and histones. Complex formation leads to decrease in molar ellipticity in the far UV CD spectra of core histones. These results led us to investigate the role of MTR as a potential epigenetic modulator. *In vitro* HAT assays using CREB-binding protein (CBP) HAT domain, a transcriptional coactivator, showed that MTR inhibits H3 K18 acetylation (specifically mediated mark by CBP/p300 *in vivo*) in H3.3 recombinant histone whereas it induces H3 K18 hyperacetylation in H3.1/H4 tetramer as well as core histones.

## Conclusions

MTR exhibits dual binding mode since it binds to histone proteins present in chromatin besides DNA [4]. It alters CBP induced H3 K18 acetylation. In contrast to MTR-DNA interaction, association of MTR with histones does not require obligatory presence of bivalent metal ions. The interaction of MTR with histones hints at a different scenario in MTR-chromatin interaction. Experiments are in progress to address the effect of MTR on other epigenetic marks. The significance of these *in vitro* experiments would also be examined by performing cell line based experiments. These data would be useful in understanding the mechanism of action of anticancer drugs as well as in designing new-generation therapeutics.
